# The Rising Popularity of Growth Hormone Therapy and Ensuing Orthopedic Complications in the Pediatric Population: A Review

**DOI:** 10.3390/children11111354

**Published:** 2024-11-07

**Authors:** Samuel Zverev, Zachary M. Tenner, Carlo Coladonato, Meredith Lazar-Antman

**Affiliations:** 1 New York University Grossman Long Island School of Medicine, Mineola, NY 11501, USA; zachary.tenner@nyulangone.org (Z.M.T.); carlo.coladonato@nyulangone.org (C.C.); meredith.lazar2@nyulangone.org (M.L.-A.); 2 Department of Orthopedic Surgery, NYU Langone Hospital—Long Island, Mineola, NY 11501, USA

**Keywords:** pediatric orthopedic surgery, orthopedic surgery, sports medicine, pediatric trauma, epiphysis, growth plate, children’s fractures, sports injuries, playground injuries, growth hormone therapy, pediatric endocrinology, endocrinology

## Abstract

The utilization of recombinant human growth hormone therapy in pediatric populations, originally approved to treat diseases of growth hormone deficiency, has expanded to encompass a broader range of indications, leading to a threefold increase in its utilization in the last two decades. However, concerns regarding its safety, particularly those that are orthopedic in nature, have grown alongside its increasing popularity. Growth hormone usage has been reported to predispose patients to a multitude of common orthopedic conditions, including carpal tunnel syndrome, Legg–Calve–Perthes disease, little league shoulder, Osgood–Schlatter disease, osteochondritis dissecans, scoliosis, Sever’s disease, and slipped femoral capital epiphysis. The pathways by which growth hormone therapy can precipitate orthopedic pathology has been shown to be multifactorial, involving mechanisms such as hormonal changes, growth plate instability, rapid growth, and increased susceptibility to overuse injury. This review examines the orthopedic consequences of growth hormone therapy in pediatric patients by discussing these potential pathophysiologic mechanisms of injury and analyzing subsequent clinical manifestations. By examining processes underlying these complications, we highlight the need for orthopedic surveillance and management in children receiving GHT, particularly those with pre-existing musculoskeletal comorbidities or high levels of physical activity. Our findings underscore the importance of a multidisciplinary approach involving co-management by pediatricians, endocrinologists, and orthopedic surgeons to optimize safety and outcomes for these patients. Directions for future research include correlating pathophysiologic mechanisms to injury patterns, investigating long-term complications in recently approved growth hormone therapy indications, and informing clinical guidelines on the management of orthopedic injuries in this patient population.

## 1. Introduction

Recombinant human growth hormone (GH) initially received approval from the United States Food and Drug Administration for the treatment of pediatric GH deficiency in 1985. Thereafter, indications for the use of recombinant human growth hormone therapy (GHT) have expanded, with only 2 of 8 being due to a finite deficiency in GH–GH deficiency and Prader–Willi syndrome. The other 6 indications, idiopathic short stature, small for gestational age, Turner syndrome, SHOX gene haploinsufficiency, Noonan syndrome, and chronic renal insufficiency, do not target GH deficiency and instead aim to augment height by supplementing endogenous GH production [1].

Short stature is one of the most common chief complaints for treating a patient with recombinant human GHT. Treatment involves daily subcutaneous injections meant to mirror the physiologic secretion of GH by the pituitary gland with the goal of modulating development primarily during adolescence [2]. It is expensive, with median costs exceeding $25,000 annually per child. Nevertheless, recombinant human GHT use in the United States’ pediatric population has tripled in the last two decades and continues to steadily increase [3]. This growth in popularity has led to increased scrutiny about its safety and subsequent unresolved safety concerns [4].

Pediatric orthopedic complications have been described to be associated with the use of recombinant human GHT. In 2011, Haidar et al. reviewed the relationships between GHT and common orthopedic injuries in pediatric populations, including carpal tunnel syndrome (CTS), Legg–Calve–Perthes disease (LCPD), scoliosis, and slipped capital femoral epiphysis (SCFE) [5,6]. Since then, novel research has continued to investigate the pathogenesis and association of recombinant human GHT and ensuing orthopedic complications [7]. Coupled with the burgeoning popularity of recombinant human GHT in the United States in the last decade, this presents a salient subject for review. In this paper, we will provide an update of the orthopedic complications examined by Haidar et al. as well as little league shoulder (LLS), Osgood–Schlatter disease, osteochondritis dissecans, and Sever’s disease. More specifically, we seek to (i) highlight the orthopedic complications of GHT regarding specific orthopedic injuries, (ii) analyze the mechanisms by which GH may be predisposing pediatric patients to injury, and (iii) demonstrate the utility of pediatric orthopedic management in adolescent patients receiving recombinant growth hormone treatment. We hypothesize that our review of the literature will demonstrate the underemphasized risk of orthopedic injury in patients on GH treatments and subsequently support a more concerted effort to manage these patients through a multidisciplinary approach.

## 2. Pathogenesis

The orthopedic diseases reviewed in this paper can be grouped by mechanism of injury to better understand the unique pathogenic consequences of GHT. CTS, LLS, Osgood–Schlatter disease, and Sever’s disease can be sorted into the overuse/repetitive stress injury category, LCPD and SCFE into the avascular necrosis/osteonecrosis category, and scoliosis and osteochondritis dissecans into the developmental abnormalities category.

The relationship between GHT and overuse/repetitive stress injury is twofold. For the pathophysiological aspect, GH has been shown to exhibit negative biochemical consequences, potentially precipitating overuse injuries and decelerating healing. In a study investigating the use of subcutaneous GH in the recovery from acute tendon-to-bone interface injuries in rat models, use of GH pre- and post-operatively demonstrated lower loads to ultimate failure and higher risk of bone fracture failure compared with the placebo group [8]. For the environmental aspect, the use of GHT in adolescent athletes may motivate them to participate in or allow them to qualify for more competitive and physically demanding levels of their sport. The correlation between incidence and risk of overuse injuries and higher level of play in adolescents is well demonstrated in the literature [9].

Avascular necrosis and osteonecrosis are complications of GHT thought to be caused by effects on bone structure, chondrocyte proliferation, and bone regeneration processes. The local stimulation of insulin-like growth factor-1 (IGF-1) by GH stimulates longitudinal bone growth and production of chondrocytes and skeletal muscle [10]. Application of GH and IGF-1 at the epiphyseal growth plate has been shown to improve fracture healing and stimulate longitudinal bone growth in rat models [11,12]. A related study in horse models demonstrated cyclic IGF-1 gene expression during spontaneous repair of acute articular cartilage injury potentially reflecting healing and chondrogenesis [13]. Dysregulation of these processes due to the introduction of growth hormone has been shown to weaken the epiphyseal plate due to hyperproliferation of chondrocytes and may lead to greater risk for subsequent injury [14]. At the microscopic level, abnormal growth hormone levels have been associated with atypical ossification nuclei and abnormal vascular architecture, leading to higher susceptibility to ischemic insults and the development of osteochondritis dissecans lesions [15].

**Figure 1:**
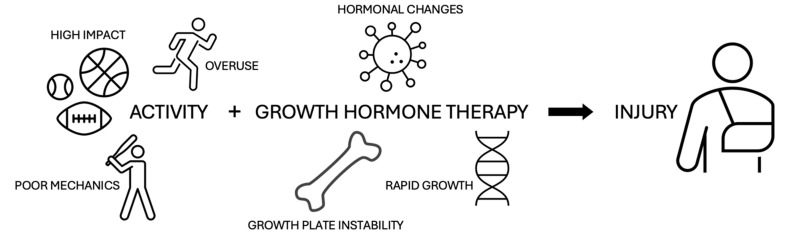
Illustrative representation of potential factors influencing injury in pediatric patients on growth hormone therapy.

The pathogenesis of GHT and its interaction with developmental disorders in adolescents is similar to that of its mechanism in osteonecrotic disorders. GH, and its harmonious relationship with IGF-1 and androgen hormones, is crucial for appropriate growth. Notably, diseases like scoliosis, which has been shown to worsen during natural stages of development characterized by increased growth, are at greater risk, as GH further accelerates growth [16]. A combination of environmental and biological factors influences the incidence of injury in pediatric patients taking GHT, especially in those who have pre-existing conditions and in those who are athletes (Figure 1).

## 3. Scoliosis

Adolescent idiopathic scoliosis, the most common type of scoliosis, is postulated to be due to hormonal causes, asymmetric growth, muscle imbalance, and/or genetic factors [17]. The risk of scoliosis curve progression is significantly correlated to rapid skeletal growth, motivating research into potentially increased risk in patients receiving GH therapy [18].

Haidar et al. reviewed this association, initially citing conflicting evidence of scoliosis as a complication of GHT but ultimately referencing the NCGS and KIGS trials, large multi-center national and international databases, respectively, both of which showed scoliosis to be a significant adverse event. A 2023 KIGS trial report, which included additional follow-up, reported scoliosis to be the second most common adverse event of GH treatment. As in the original paper, patients being treated for Prader–Willi syndrome were seen to suffer the highest incidence of scoliosis [19,20].

A recent independent cross-sectional and retrospective cohort study investigated scoliosis incidence in patients being treated with GHT for idiopathic short stature. The study found that among patients with idiopathic short stature without scoliosis at baseline, treatment with GH significantly increased the risk of developing scoliosis and the need for bracing [16]. Despite the emergence of conflicting data, with another recent study showing no association between the administration of GHT and the development of scoliosis [21], these findings indicate that a multidisciplinary team, which includes orthotists, healthcare professionals who design and fit orthoses (i.e., braces and splints), is important for the management and prophylaxis of potential scoliosis development in this patient population.

## 4. Osteochondritis Dissecans

Osteochondritis dissecans is defined as a focal alteration of subchondral bone with risk of instability and disruption of adjacent articular cartilage. This phenomenon puts effected joints at risk of premature osteoarthritis, especially in the presence of articular cartilage fracture or subchondral bone separation. The mechanism is not fully understood and is difficult to study, as osteochondritis dissecans often presents when clinical symptoms are overt. The goals of treatment lie mainly in symptom management and joint preservation. The effects of GHT on the susceptibility to damage and separation of the joint surface and growth plate may play a role in its intersection with the development of osteochondritis dissecans [22].

In one case study, a young male patient being treated with GHT for acquired human GH deficiency presented with multiple osteochondritis dissecans lesions over the course of a year in his left medial femoral condyle, left lateral femoral condyle, and right lateral humeral condyle [23]. The occurrence of multiple osteochondritis dissecans lesions and an asymmetric nature of these lesions occurring is rare [24]. This rarity, combined with the effect of GHT on physeal structure, led the authors to implicate GHT in the pathogenesis of this patient’s disease.

Another similar case study described multi-joint osteochondritis dissecans approximately six months after starting GHT. The patient, also a young male, suffered lesions in his elbows, knees, and ankles that limited daily physical functioning. Treatment with GH was stopped one year after initiation, and the patient underwent bilateral knee arthroscopy and osteochondritis dissecans micro-drilling [25]. Like in the case study discussed previously, the rare occurrence of osteochondritis dissecans lesions in more than one joint and the recent initiation of GHT implicate it as a salient culprit in the pathogenesis of the patient’s presentation. Additional high-quality studies are required to establish the potential relationship between osteochondritis dissecans and the use of GHT.

## 5. Slipped Capital Femoral Epiphysis

SCFE is characterized by an external rotation of the metaphysis with anterior translation and slippage while the epiphysis stays in the acetabulum and is hypothesized to be due to a high physiologic axial load transmitted across a relatively weak physis [26]. It is the most common hip pathology in pre-adolescents and adolescents, affecting approximately 11 out of every 100,000 patients [27,28]. Known risk factors for SCFE include obesity, endocrinopathy, male sex, periods of rapid growth, radiation, and mechanical shearing forces to the area, among others [29]. Research into the association between SCFE and hormonal derangements has recently shown GH deficiency to be associated with the highest incidence of SCFE among children with endocrinopathies, with approximately 584 out of every 100,000 patients going on to develop the condition [30].

Haidar et al. reviewed this association, citing the identification of GHT as a risk factor as early as the 1970s. GHT in the settings of idiopathic GH deficiency, idiopathic short stature, Turner syndrome, organic GH deficiency, end-stage renal disease, kidney transplant, and dialysis all reported higher incidence of SCFE compared with the general pediatric population [31,32,33,34].

Novel studies have continued to cite associations between SCFE and GHT. In one case study, a young female patient with Turner syndrome on one year of GHT, who initially presented with a limp, was found to have bilateral SCFE. Although SCFE has been shown to be a sequalae of both GH deficiency and GHT, Mona et al. emphasized the onset of this patient’s SCFE during her course of GHT specifically [35]. Their findings are supported by existing literature that showed a tenfold greater incidence of SCFE in children with Turner syndrome when compared to all children and a 24-fold greater incidence in children with Turner syndrome on GHT when compared to all children [36]. These findings suggest that the use of GHT may contribute to the development of SCFE in patients with Turner syndrome.

A recent study showed that childhood cancer survivors exposed to total body irradiation with subsequent irradiation-induced GH deficiency were seen to have significantly greater risk for SCFE development during recombinant GHT. The rate of SCFE in children treated with recombinant GHT for irradiation-induced GH deficiency was shown to be 211-fold greater than reported in children treated for idiopathic GH deficiency [37]. These findings suggest that the combination of radiation exposure and GHT puts children at an exponentially increased risk for developing SCFE.

Mittal et al. conducted a cohort analysis involving 36,791 pediatric patients receiving GHT, ultimately demonstrating that patients on GHT had a significantly increased risk of developing SCFE, compared to controls, in a dose-dependent manner. Authors recommended preemptive screening for SCFE during clinical visits involving questions about hip and knee pain for physicians and parents and a high index of suspicion for SCFE if these symptoms may arise [38].

## 6. Legg–Calve–Perthes Disease

LCPD, most frequently observed in pediatric males (aged between two to 12 years), is identified as avascular necrosis occurring due to the loss of circulation to the capital femoral epiphysis. Although most cases of LCPD are described to be idiopathic, osteonecrosis can occur due to a variety of factors, including trauma, coagulopathy, exposure to cigarette smoke, varied endocrine function, and chronic renal insufficiency [5,39]. Trends in pediatric epidemiology have been shown to affect the incidence of LCPD. Most recently, researchers out of the United Kingdom mapped decreasing rates of pediatric exposure to cigarette smoke to decreased incidence of LCPD [40]. This relationship motivates us to evaluate another trend, the increased prescription of exogenous GH and its potential association with the development of LCPD [3].

To better describe the relationship between GH and LCPD, Lamback et al. recently performed a retrospective analysis of pediatric and adolescent patients with hip disorders and GH deficiency in London from 1992 to 2018. Of the three patients identified for LCPD, two had developed LCPD after beginning GHT, while one three-year-old male was diagnosed with LCPD before starting GHT one year later [41]. In the child that developed LCPD before starting GHT, the GHT halted its progression. Hinting at a potential protective effect of GHT for patients diagnosed with LCPD, Shi et al. presented a similar case study of a pediatric patient that experienced similar curative effects of Perthes’ disease after four years of GHT [42]. These cases present the potential beneficial effect of GHT in children with LCPD.

With regard to the other two pediatric patients Lamback et al. presented, there have been cases presenting the negative, more causative effects that GH therapy may have on the development of LCPD [41]. Lim et al. presented a case study of an eight-year-old female with skeletal dysplasia that was treated for short stature with GHT then 7 months later developed LCPD [43]. Due to the varied observed cases in the recent decade, IGF-1 has been evaluated as an effective factor in the etiology of LCPD [44]. The exact effect and mechanism of the relationship between GHT and LCPD development has not been established, but patients should be monitored for LCPD development before and during treatment with GHT.

## 7. Little League Shoulder

LLS, also known as proximal humeral epiphysiolysis, occurs most often in high-performance adolescent baseball pitchers, as well as in young athletes with poor mechanics. These physeal stress fractures most often occur to children between the ages of 11 to 16 years because of the fragility that exists within these regions during periods of rapid growth [45]. As a result, adolescents are more susceptible to these injuries, especially as they compete at high levels during their growth spurt. They are especially susceptible in situations where children are required to pitch from professional distances of 60 feet 6 inches, rather than 46 feet, thus causing increased disruption to chondrocytes closest to the epiphysis in the zone of proliferation. These cells, which are under the influence of GH, may have a decreased ability to produce strong cartilage matrix in these individuals [46]. Pediatric and adolescent athletes that use GHT may have increased rates of LLS injuries as their utilizations increase.

The Little League Baseball industry has exploded over recent decades, as middle- and high-school athletes tend to focus their energy on a single sport over the course of the year. Boston Children’s Hospital identified an increased in the incidence of LLS of approximately 8% per year on average from 1999 to 2013 [47]. As LLS is being diagnosed with increasing frequency, GHT is also being increasingly used in these same pediatric athlete populations. There is a recent case report of a 16-year-old male baseball player who sustained LLS in the context of being treated with human GH treatments [48]. Although GH is not an established etiology for growth plate cartilage fractures, the pathophysiology and causative nature behind GH and these pathogenic fractures requires further investigation as the incidence of LLS increases.

## 8. Osgood–Schlatter Disease

Osgood–Schlatter’s disease is a condition causing painful inflammation of the tibial tubercle physis, most commonly occurring in adolescent patients undergoing rapid growth [49]. The incidence of Osgood–Schlatter disease is known to affect about 1 in 10 athletic adolescents, depending on factors including the shortening of the rectus femoris muscle, the patient’s degree of development, and the regular practice of sports in the pubertal phase [50,51] Although physical activity has been linked to the development of Osgood–Schlatter disease, moderate physical activity is also known to benefit adolescents through increased GH activity [52,53]. These consequences of physical activity complicate the relationship between Osgood–Schlatter disease and GHT because an argument can be made for both its protective and destructive pathogenicity.

The pathogenesis of Osgood–Schlatter disease has been related to pain from both underlying tendinous injury and cartilaginous avulsion, depending on the mechanism of injury [50]. Beber et al. identified a strong relationship between tibial avulsion fractures and GHT in the pediatric population [7]. In the case–control study, authors discovered that GHT at the time of injury was “significantly greater than that of age, sex, and body mass index-matched control group with midshaft tibial fractures”, demonstrating that it is a potential orthopedic complication of GHT treatments.

Similarly, Brown et al. reported that during periods of rapid growth, where Osgood–Schlatter disease risk is highest, the growth plate is more fragile than the surrounding ligamentous and tendinous tissue [46]. If further research demonstrates that rates of Osgood–Schlatter disease are higher in pediatric patients treated with GHT, it would be reasonable to consider avulsion fractures as a more direct cause of Osgood–Schlatter disease than tendinous injury. On the other hand, if there is no link, it would strengthen the idea of tendinous and ligamentous injury as being more closely related to Osgood–Schlatter disease.

Recent literature has demonstrated a significantly higher incidence of tibial tubercle fractures among patients with OSD, potentially due to a similar mechanism of injury [54]. The use of GHT has been identified as a potential risk factor for tibial tubercle fractures due to a negative impact on the strength of growth plates and increased fracture risk [55]. This relationship further implicates concomitant GHT use in the pathogenesis of these orthopedic injuries. Regardless, further research into the incidence rates of GHT and the pathogenesis of Osgood–Schlatter disease is required to guide preventative patient care and treatment.

## 9. Sever’s Disease

Sever’s disease, or calcaneal apophysitis, is the result of an overuse injury due to traction apophysitis at the Achilles tendon insertion site on the calcaneus [56]. It is a common cause of heel pain in skeletally immature athletes, usually occurring during rapid growth or sudden increase in athletic activity [57]. The particular susceptibility of young athletes to Sever’s disease is due to inability of the muscle–tendon unit to stretch sufficiently during rapid bone growth, resulting in increased tension across the unossified or incompletely ossified apophysis [58]. The increase in GH use in the pediatric population and the control GH exerts on bone development make its potential to cause Sever’s disease a salient complication to investigate [3,59].

One recent case study described a young male patient with isolated GH deficiency in his first year of GHT who presented with pain in both heels and limping subsequently diagnosed with bilateral Sever’s disease and no history of trauma or athletic overuse. The development of Sever’s disease in this patient shortly after beginning GHT and without typical risk factors, such as biomechanical stress and obesity, led the authors to implicate GHT in the pathogenesis of his presentation. This finding prompts the consideration of Sever’s disease as a potential orthopedic complication of GHT [60].

## 10. Carpal Tunnel Syndrome

Carpal tunnel syndrome involves the compression of the median nerve by the transverse carpal ligament in the wrist [61]. Common causes of CTS include overuse, anatomical factors, hormonal changes, and comorbidities that affect joints and glycemic control such as diabetes. The effect of GHT on endocrine and inflammatory mechanisms and its relationship with the development of diseases of glycemic control warrant the evaluation and update of CTS development in the setting of GHT [62].

Since Maneatis et al. reported the low incidence of 27 per 100,000 patients receiving GHT developing CTS in 2000, the literature has shown significant increases in CTS in individuals receiving hormonal replacement therapy and in those with acromegaly, a state of endogenous GH overproduction [63,64,65]. The majority of CTS cases that present in pediatrics are related to genetic factors [66]. The pediatric cases of CTS have commonly been reported in relationship to lysosomal storage disease documented in case reports about fibrillin-1 gene mutations [67]. Despite the proposed relationship between GHT and CTS, there has not been an updated incidence rate since the 1996 National Cooperative Growth Study estimate of 27 per 100,000 in all patients [68].

A potential side effect in children receiving GH has been the development of insulin resistance and disorders of glucose tolerance that may lead to hyperglycemia [69]. In these cases, researchers recommend formal monitoring of glucose metabolism in GH recipients predisposed to an increased risk of developing diabetes [70]. CTS has been shown to have higher incidences in diabetic populations, even when compared to hypothyroid and acromegaly groups [71,72]. The risk of developing a disease of glucose metabolism in the setting of GHT and the increased incidence of CTS in the diabetic population proposes a theoretical mechanism by which GHT may predispose pediatric patients to CTS.

## 11. Discussion

A large body of literature published over nearly four decades has demonstrated the efficacy of GHT for providing short-term height gain in patients with both GH-deficient and non-GH-deficient conditions [20]. Benefits of GHT, including enabling patients to reach adult heights near the normal range, have been established in diseases like Turner syndrome, for which growth hormone therapy has been FDA approved since 1996 [73]. This proven efficacy of GHT has allowed its indications to continue to expand, most recently with Noonan syndrome in 2007, for which it has also been shown to be effective [74]. As the treatment becomes approved for more conditions affecting growth, further longitudinal studies will be needed to assess long-term complications.

The escalating utilization of GHT within the United States population further underscores the importance for comprehensive investigation into its potential complications, including those that are orthopedic in nature. Motivations behind GHT administration often stem from indications other than GH deficiency, such as from aspirations for physical enhancement, with short stature being the most prevalent concern [1]. In this comprehensive review, we explored the relationship between GHT and a spectrum of orthopedic conditions, including CTS, LCPD, LLS, Osgood–Schlatter disease, osteochondritis dissecans, scoliosis, Sever’s disease, and SCFE.

Management of orthopedic complications of GHT are patient- and complication-specific. Treatment is twofold, involving amelioration of the new orthopedic injury and planning regarding the patient’s GHT. Treatment of the insult will involve moving along the treatment algorithm as with a patient who is not taking GHT. This means monitoring, bracing, and/or operating on a patient who develops scoliosis and surgical pinning in a patient who develops SCFE regardless of GHT treatment status [6]. GHT planning may involve dose modification, treatment interruption, or, in rare cases, permanent discontinuation if symptoms do not improve.

Although a variety of sources that demonstrate the orthopedic complications of GHT in pediatric patients was discussed, this review is challenged by the existence of compelling bodies of conflicting literature. Research describing both no association, exemplified by a 2022 study showing no effect of GH treatment on scoliosis development or aggravation, and research describing associated benefit, with a 2023 study showing halting of LCPD progression with the use of GHT, motivate analyses weighing the risks and benefits of GHT in individual patients [21,42]. The nascent understanding of the pathophysiologic mechanisms behind GHT’s orthopedic complications further complicates this decision and motivates further research. Other limitations of our review include its scope, as we primarily concentrated on the most common pediatric orthopedic pathologies, consequently excluding more rare conditions, and lack of experimental validation, as our analysis is based on existing literature rather than empirical data. Additionally, this is a traditional review rather than a systematic review, meaning some studies within our scope may have been missed.

## 12. Conclusions

While GHT harbors therapeutic promise for a myriad of pediatric conditions, the imperative to scrutinize its potential adverse effects requires clinical attention. Subsequent investigations should endeavor to determine the precise pathogenesis of GHT’s orthopedic complications, and concerted efforts are warranted to mitigate these negative sequelae. Prospective pediatric patients evaluated for the implementation of GHT, particularly those who have musculoskeletal comorbidities or who are athletes, should undergo diligent monitoring by a multidisciplinary healthcare team, which includes primary care pediatricians, pediatric endocrinologists, and pediatric orthopedic surgeons, to preempt and address any significant complications that may arise.
